# An Overview of Microbial Fuel Cell Technology for Sustainable Electricity Production

**DOI:** 10.3390/membranes13110884

**Published:** 2023-11-17

**Authors:** Wilgince Apollon

**Affiliations:** Department of Agricultural and Food Engineering, Faculty of Agronomy, Autonomous University of Nuevo León, Francisco Villa S/N, Ex-Hacienda El Canadá, General Escobedo 66050, Nuevo León, Mexico; wilgince.apollon@uanl.edu.mx or apollonwilgince@gmail.com; Tel.: +52-812-153-2628

**Keywords:** bioelectrochemical system, bioenergy, fuel production, microbial fuel cell

## Abstract

**Highlights:**

- Configurations and operations of microbial fuel cells are discussed.
- Bioelectrochemical system performance depends on the type of design and electrode materials.
- Microbial fuel cells are a feasible alternative for fuel production and wastewater treatment.
- Advances in electrode materials are summarized.
- Techno-economic and life-cycle assessments of microbial fuel cells are highlighted.

**Abstract:**

The over-exploitation of fossil fuels and their negative environmental impacts have attracted the attention of researchers worldwide, and efforts have been made to propose alternatives for the production of sustainable and clean energy. One proposed alternative is the implementation of bioelectrochemical systems (BESs), such as microbial fuel cells (MFCs), which are sustainable and environmentally friendly. MFCs are devices that use bacterial activity to break down organic matter while generating sustainable electricity. Furthermore, MFCs can produce bioelectricity from various substrates, including domestic wastewater (DWW), municipal wastewater (MWW), and potato and fruit wastes, reducing environmental contamination and decreasing energy consumption and treatment costs. This review focuses on recent advancements regarding the design, configuration, and operation mode of MFCs, as well as their capacity to produce bioelectricity (e.g., 2203 mW/m^2^) and fuels (i.e., H_2_: 438.7 mg/L and CH_4_: 358.7 mg/L). Furthermore, this review highlights practical applications, challenges, and the life-cycle assessment (LCA) of MFCs. Despite the promising biotechnological development of MFCs, great efforts should be made to implement them in a real-time and commercially viable manner.

## 1. Introduction

In response to the environmental pollution caused by conventional energy sources, researchers worldwide have searched for reliable and sustainable energy production sources. In this line, bioelectrochemical systems (BESs) capable of transforming chemical energy into electrical energy aided by microorganisms as catalysts have been developed [1]. BESs are promising technologies that have been implemented to replace energy sources from fossil fuels, such as petroleum, natural gas, and coal [1,2], in response to worldwide high energy demands. BESs can be classified into (i) microbial desalination cells (MDCs) [3,4,5], (ii) microbial electrosynthesis cells (MECs) [6], (iii) enzymatic biofuel cells (EBCs) [7], (iv) electrolysis cells (ECs) [8], (v) microbial solar cells (MSCs) [9], (vi) biobatteries [10,11], (vii) constructed wetland microbial fuel cells (CW-MFCs) [12], and (viii) microbial fuel cells (MFCs). To date, considering the types of BESs, MFCs are the oldest, being the first BES presented in 1911 [13]. The operation of an MFC is based on bacterial activity, where so-called electrochemically active bacteria (EAB) break down (i.e., oxidize) organic matter (OM) to produce bioelectricity [14]. During this process, the EAB use their metabolic pathway to transport electrons [15,16].

**Figure 1:**
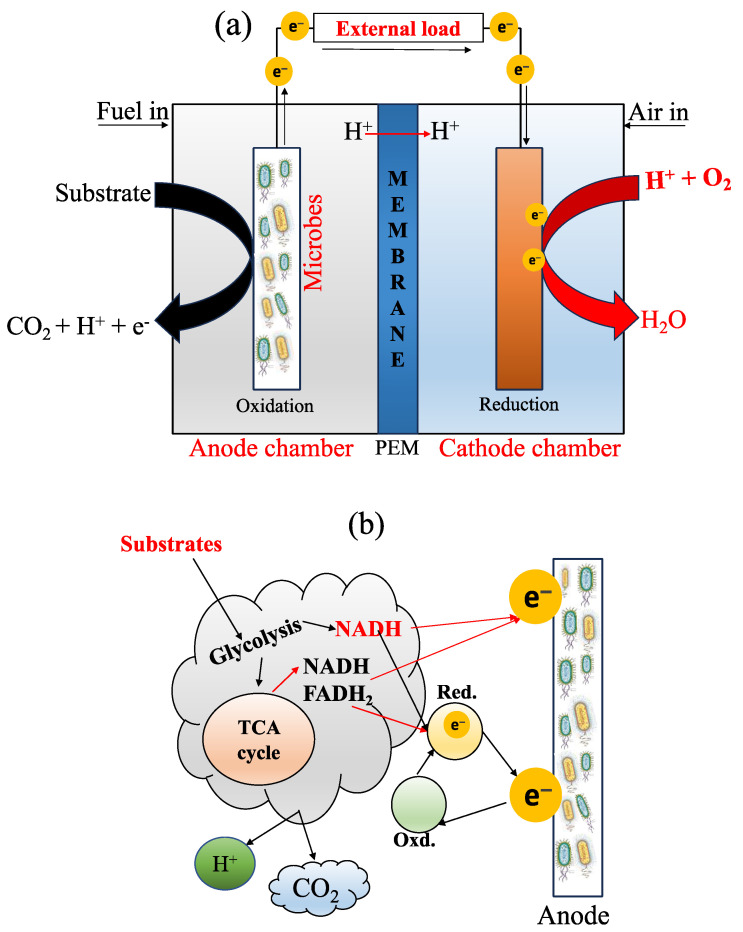
(**a**) A typical diagram of an MFC with a PEM (proton exchange membrane) and its bioelectrogenic process; and (**b**) the electron transport mechanism, adapted from [33]. During the process, there are three parameters to consider: the microorganism’s structures to carry out the phenomenon, the type of microorganism, and the electrical conductivity of the anode material. So far, there are three methods for electron transfer through EAB activity: (i) electron transfer through redox-active protein molecules, (ii) the use of shuttle electrons to transfer electrons, and (iii) direct electron transfer through conductive pili [34]. When the material has high conductivity, it helps to improve the flow of electrons, exhibiting less resistance [35]. In addition, the mechanism of electron transfer also involves natural mediators, directors of electron transfer, and synthetic mediators [19].

The components of an MFC basically include an anode compartment and a cathode compartment (Figure 1a), both separated internally by a membrane. The anode chamber is mainly responsible for the oxidation of OM (Equation (1)) in the substrate (e.g., in presence of water) through microbial activity, producing electrons (e^−^), protons (H^+^), and carbon dioxide (CO_2_) [17]; then, e^−^ are transported toward the cathode chamber [14,18]. Subsequently, the protons are associated with oxygen, thanks to the PEM (Equation (2)), in order to form an essential compound in the reaction: the water molecule (H_2_O). Equation (3) indicates the complete chemical reaction in an MFC. Additionally, the electron transfer mechanism at the anode surface of an MFC is presented in Figure 1b. The author referred to the following critical reviews to better understand this area [16,19].

(1) $${\mathrm{Anode}:C}_{6}H_{12}O_{6}+6H_{2}O\to 6CO_{2}+24H^{+}+24e^{-}$$

(2) $${\mathrm{Cathode}:O}_{2}+{4H}^{+}+4e^{-}\to 2H_{2}O$$

(3) $${\mathrm{Overall}\;\mathrm{reaction}:C_{6}H_{12}O_{6}+6H_{2}O+6O}_{2}\to 6CO_{2}+12H_{2}O$$

Since the implementation of MFCs, wastewater has been used as an excellent organic substrate for these systems, improving their efficiency in both bioenergy generation and waste management [20,21,22,23,24,25]. The highest power density of 4.99 ± 0.02 W/m^2^ was reached in a dual-chamber MFC inoculated with an excellent mixed culture [26]. This high performance was obtained in a three-dimensional N-doped bio-anode MFC fabricated with carbon felt as the anode and cathode and was higher than that of previous studies using carbon-based materials. The MFC reactor had a working volume of 80 mL in each chamber. The system’s high performance was likely due to the electrode materials used. More recently, a high power density of 1793 ± 77 mW/m^2^ was achieved in an MFC operated with atomically dispersed Fe–N_4_ moieties as an excellent cathode catalyst [27]. The atomically dispersed Fe–N_4_ moieties were considered a perfect option to enhance the performance of the MFCs.

Additionally, the performance of an MFC system depends mainly on the electrode materials, anolyte/catholyte, pH of the medium, bacterial communities [17], type of substrate, configuration, and operating conditions, as has been critically reviewed in the literature [28,29,30]. Furthermore, factors such as chemical oxygen demand (COD) and fuel concentration, membrane thickness, and operating temperature also affect the performance of MFCs [31,32]. Section 2 discusses the factors of MFC technology, including MFC designs, configurations, and operation. The development of MFCs could provide excellent alternatives for low-income countries, being a cheap biotechnology compared to traditional energy sources. Even though MFCs face significant challenges, they remain an ecological and economical option worldwide. This review is quite comprehensive, compared to other studies, as it presents recent advances in the factors influencing the performance of MFCs, including the design type, configuration and operation, electrode materials, membranes, the influence of microorganisms, and substrates. In addition, this review deeply discusses real-time applications, challenges, and life-cycle assessments (LCAs) of MFCs, considering previous reviews [2,4,5,15] that did not perform an LCA simultaneously. Finally, the review outcomes have strong implications, and key contributions to the field are discussed below, particularly emphasizing that MFCs are the only devices that can convert biodegradable organic matter into electrical energy without requiring an additional energy source.

## 2. Influence Factors of MFCs

### 2.1. Designs, Configurations, and Operation

**Figure 2:**
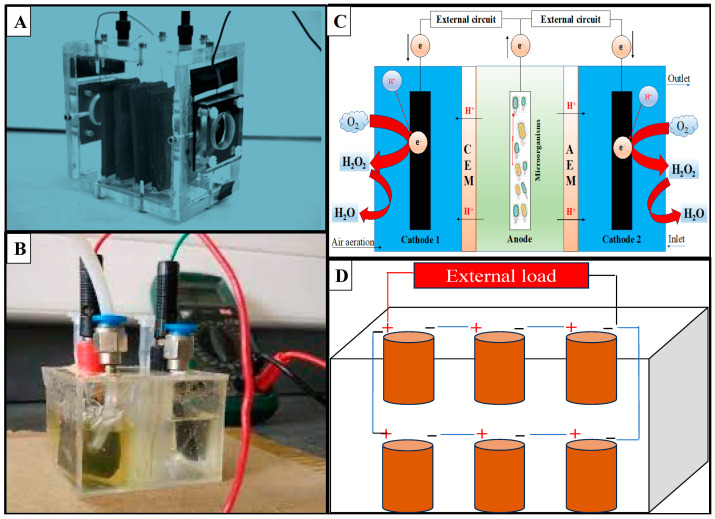
Designs, configurations, and schematics of MFCs previously presented at lab scale: (**A**) SC-MFC [43]; (**B**) DC-MFC inoculated with *Escherichia coli* strain [44]; (**C**) triple-chamber MFC; and (**D**) stacked MFCs.

Since the implementation of MFCs, different types of designs and configurations have been proposed to improve their efficiencies, including single-chamber MFCs (SC-MFCs; Figure 2A), dual-chamber MFCs (DC-MFCs; Figure 2B), triple-chamber MFCs (TC-MFCs; Figure 2C) or more, and stacked MFCs (Figure 2D). SC-MFCs and DC-MFCs are the most commonly used BESs in this field. However, stacking MFCs can increase the voltage output significantly, as has been reported in a recent critical review [36].

Furthermore, much research concerning the design and configuration of MFCs has focused on improving the performance of these systems by modifying the anode surface area [37,38,39,40,41]. This process involves factors such as conductivity, and the materials used alter the electrode. Modifying the anode electrode provides an excellent option to improve power generation and electron transfer, which is a crucial factor affecting the performance of MFCs. The anode is the engine in a BES, as it is the only region where the EABs act as biocatalysts and oxidation impellers to generate electricity.

**Figure 3:**
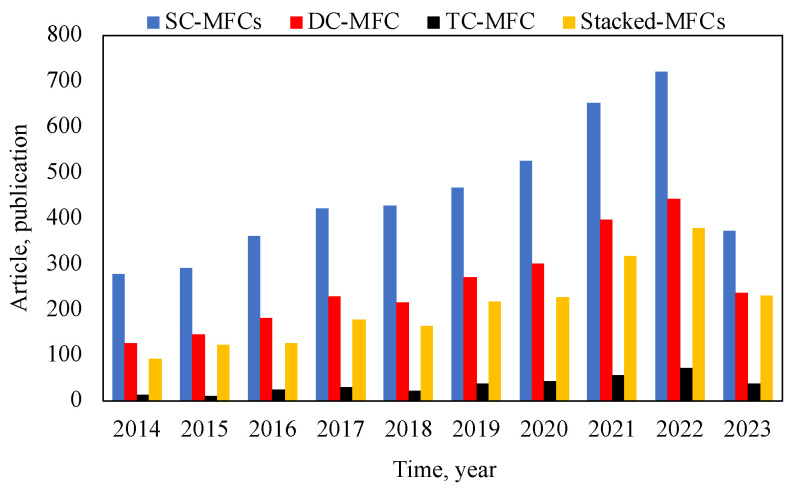
The number of articles on different designs and configurations of MFCs from 2014 to 2023 (Source: ScienceDirect database; 1 June 2023).

Many studies on different types of MFC designs have been carried out over the last decade. According to the ScienceDirect database (up to 1 June 2023), a total of 9470 research articles have been published in different Elsevier journals, of which 4519 focused on SC-MFCs, 2546 on DC-MFCs, 2054 on Stacked-MFCs, and 351 on TC-MFCs. Figure 3 presents the different types of MFC configurations reported in previous studies. It can be seen that the number of studies per year increased from 2014 to 2023, and much effort has been put into improving the operation and performance of this technology. There were more studies on SC-MFCs than the other MFCs as they are cheaper to set up, easy to use, have low internal resistance, have greater proton diffusion, and have high oxygen reduction in the cathode chamber [42] in comparison with other MFC systems. The following paragraph discusses the bioenergy production of the different types of MFCs in terms of power density, as reported in recent studies.

In one experiment by Choudhury et al. [45], two SC-MFCs with the same characteristics (identical) were designed and built with 300 mL of water (working volume). The anode and cathode were made with carbon cloth. The two chambers were dissociated with a Nafion 117 membrane. The anode and cathode were separated by a distance of 157 μm, which was considered a crucial factor in the system’s performance. To start up the system, a pure strain was added to the MFCs, and dairy wastewater was used as the primary source of inoculum. According to the authors, this was the first time that the strain *Pseudomonas aeruginosa*-MTCC-7814 had been applied in an MFC with the same characteristics. The system operated for 15 days, and the highest power density achieved was 105 mW/m^2^ with a current of 313 mA/m^2^. This performance was obtained when the SC-MFCs were connected in series, producing a maximum voltage of 1025 mV. The above results were interesting, and the authors hypothesized that adding the pure strain *Pseudomonas aeruginosa* improved power production in the SC-MFC. However, when the structure of the MFC was changed (i.e., in terms of the anode electrode material), a maximum power density of ∼104 mW/m^2^ was observed [46]. This result was statistically similar to that reported earlier [45]. Therefore, the difference between the above studies lies in the materials and type of configuration and operation of the systems. In the study by Chaturvedi et al., an SC-MFC with an air cathode was used. Furthermore, the anode and cathode were separated with Nafion 212 as a membrane. In addition, when the DC-MFCs were inoculated with *Shewanella putrefaciens* [47] and pure cultures [48], the highest power densities reached were 1190.9 and 1220 mW/m^2^, respectively, due to modification of the electrode. These results were higher than those reported in the above-discussed studies. This achievement in the SC-MFCs was due to the anode electrode modification [49,50]. The reader is referred to a previous overview for a comprehensive review of this topic [51].

The above results demonstrated that most approaches for generating electricity using BESs have been limited to laboratory research, and the scaling up of these systems for practical application still needs to be improved. Hence, commercial and real-time application use must be the focus of further progress. In addition, there is a need to scale MFC systems to a practical level, in order to understand the usefulness of the bacteria’s electroactivity concerning the sustainable recovery of electricity, organic nutrients, and value-added products from wastewater with zero contamination. However, scaling up MFC systems requires searching for cheaper and more suitable materials than conventional ones [52].

More recently, a novel SC-MFC based on a hydroponic system (MFC-Hyp), consisting of a plastic container with a practical water volume of 2500 mL, has been presented [53]. Three units consisting of an air cathode were used to evaluate both the power generation performance and plant growth parameters, as well as wastewater treatment. For the system assembly, a platinum (Pt)-coated carbon cloth (0.3 mg/cm^2^) was used to build an air-cathode electrode. Meanwhile, for the anode electrode, bamboo charcoal (four pieces connected in series) was used. Peruvian clay ceramic was used as a membrane, due to its excellent surface porosity. Subsequently, the air-cathode MFC-Hyps with different working volumes were operated in a batch mode at room temperature (in a lab-scale experiment) for 49 days. With the idea of evaluating the system’s performance, MFC-Hyps were assessed with and without the presence of plants (*Allium tuberosum*). The obtained data indicated that, when the MFC-Hyp system was embedded with a plant, the power generation efficiency was improved and more stable, indicating a higher power density of 130.2 mW/m^2^ with the highest COD removal of 96.3%, compared to those without plants. Furthermore, one of the advantages of the MFC-Hyp system is its positive effect on plant growth parameters, and agricultural farmers can adopt this kind of technology for the sustainable production of aliments. Another advantage of this system is the production of organic fertilizers, such as nitrogen (N), potassium (K), and phosphorous (P), thus reducing the risk of GHG emissions into the atmosphere compared to conventional agriculture. However, more studies in MFC-Hyp systems are required to shift this technology from lab-scale (as was the case of the study by Sato et al. [53]) to practical applications. In contrast, in another study by Marassi et al. [54], dairy effluent (wastewater) inoculated with a microbial consortium (i.e., *S. oneidensis* and *C. butyricum*) was placed in an air-cathode MFC to improve the system performance. The air-cathode MFC was operated during two stages (or phases; 75 and 30 days) and showed great potential to produce power, as well as presenting high contaminant removal efficiency, with a biochemical oxygen demand (BOD) and COD of 96% and 94%, respectively, at 75 days. At the same time, the air-cathode MFC indicated high removal efficiencies of 100% for NO_3_^−^, 95% for P, 75% for sulfate, and 47% for organic N. The system’s performance was considered to be due to the consortium and efficiency of the air-cathode operating conditions. In another recent study, an SC-MFC based on an air-cathode was operated in an open field for 25 days, using urine as a substrate, and showed excellent performance regarding power generation with cow urine [55]. This performance was due to the composition of the urine and its association with the organic substrate. The above studies have shown how crucial the use of MFCs with air-cathodes is, in terms of overcoming their limitations in power generation and wastewater treatment.

On the other hand, DC-MFCs, like other types of BESs, have been implemented for wastewater treatment, elimination of contaminants in the soil, nutrient recovery for sustainable agriculture, and bioelectricity generation. Researchers have used different methods to improve these systems in terms of power efficiency, working hard to manufacture prototypes which are capable of continuously generating bioelectricity with high power. Table 1 compares the different types of configurations of MFCs proposed to improve their power generation performance. As can be seen from the table, these systems have recently been used to treat various wastes, including wastewater. High removal efficiencies (%) of uranium [U(VI)], nickel (Ni), COD, sulfide, and copper (Cu^+2^) were achieved along with the highest power density. Furthermore, in this comparative Table, each reactor’s working volume and operating time were considered.

An MFC with suitable electrode materials can power a digital clock, an LED [10], and biosensors [56] without any power or booster. These findings demonstrate the tremendous advances that have been made in this field of research for the real-time application of this technology. In a recent study [57], metal oxides (CuO, MnO_2_, and SnO_2_) were applied in a DC-MFC as cathodic catalysts in order to recover phenolic compounds and generate bioelectricity using industrial wastewater. Apart from using these types of catalysts, the system was configured with carbon felt and carbon plate as anodic and cathodic electrode materials, respectively. Once the system configuration was completed, it was operated for 168 h. The findings of this exciting study demonstrated the feasibility and potential of DC-MFCs to generate bioelectricity while removing phenolics in wastewater. It has been observed that parameters such as pH and temperature influence the performance of DC-MFCs. For example, when the catholyte was pH 8 (alkaline), the system produced a maximum power density of 29.24 mW/m^2^; in contrast, at pH 11, the bioelectricity production decreased by 43.50% (i.e., to 16.52 mW/m^2^) [57]. This study demonstrated that high alkalinity has a negative effect on the performance of BESs; however, further research on other factors influencing the performance of BESs is required.

The utility of MFCs can be expected to bring about a paradigm shift built on sustainability principles, encompassing the closed-loop biorefinery approach. Such efforts would help to manage waste and generate renewable fuel and value-added products, thus fostering sustainable development [58]. On the other hand, the use of MFC technology has excellent potential for GHG mitigation, through the direct reduction in CH_4_ emissions by MFCs installed in waterlogging and paddy fields [59], due to the reduced use of traditional energy resources through the production of bioelectricity in an alternative way. Furthermore, determination of the type of BES technology (such as MFCs) that can reach the highest power generation efficiency faces the problem of the need for a single unit to measure the generation of bioelectricity. Power generation is represented in the literature using various different units, such as mA, mV, mWm^−2^, and mWm^−3^, making these systems challenging to compare [29].

Furthermore, in another study [60], a DC-MFC with a new proton exchange membrane (polypropylene) was proposed, designed, and built. Graphite rods were used as the electrode materials for the anode and cathode. The system operated for eight days and produced a highest power density of 0.7 mW/m^2^, 97.6% lower than the bioelectricity generation reported in the previous study [58]. Due to this low performance obtained in the DC-MFC with a polypropylene membrane, it is recommended to conduct more studies using MFCs with the same characteristics to improve their performance. A good recommendation would be to double the cathode surface area and modify the electrode spacing to increase the power generation yield [61].

In summary, the above studies demonstrated that improved power generation using BES systems (i.e., MFC technologies) can be attained by coupling two inter-related systems (hybrids). Comparing the type of configuration of MFC technologies, it is not easy to say that one system is more effective than another if it presents a high efficiency rate. For example, the SC-MFCs based on an air-cathode configuration play a significant role in the implementation and performance of MFCs, considering different advantages such as robustness, low cost, simplicity in terms of structure and use, and low/less internal resistance (which makes them more efficient electrochemically), as has been previously reviewed [42,62]. However, despite the significant advantages of air-cathode SC-MFCs, they also present many disadvantages, such as the formation of precipitates on the cathode surface and the evaporation of water [53], high price (depending on the material used, e.g., Pt), large-scale application limitations, and biofouling issues over time [63]. Previous studies have demonstrated that the DC-MFC configuration can overcome the limitations of the SC-MFC configuration by reducing oxygen diffusion. Such a result was obtained using a separating membrane without affecting the power density and the internal cell resistance, as described in [64]. The main advantages of DC-MFCs are their capacity to produce high output power, voltage, and 100% reduction efficiency of heavy metals, such as hexavalent chromium Cr(VI), from municipal/industrial wastewater [64,65]. However, like SC-MFCs, DC-MFCs also have significant disadvantages. The main disadvantage of this type of configuration is that the cathode chamber (including the solution cathode) needs to be constantly aerated to provide O_2_ in the cathode chamber [66]; when the cathode is less/not aerated, the power density decreases significantly. According to the above, it can be argued that the performance of an MFC system also depends on the choice of electrode materials and operating conditions. In addition, improved MFCs could also be obtained by adding redox mediators and modifying the contained microorganisms, which may improve the electron transfer efficiency of the system [67].

**Table 1:** Recent developments of MFCs in terms of design and configuration.

Configuration of BES	Working Volume (mL)	Operation (Days)	Type of Electrolyte	Removal Efficiency (%)	Maximum Power Generation	Refs.
SC-MFC	850	~30	Activated sludge	N/A	105 mW/m^2^	[61]
SC-MFC	150	30	Synthetic wastewaters	89 (COD)	450.36 mW/m^2^	[68]
SC-MFC	80	N/A	Wastewater	83 (COD)	548 mW/m^2^	[69]
SC-MFC	N/A	18	Wastewater	81; 94 (COD)	989 mV	[70]
SC-MFC	100	N/A	Wastewater	73.7 HCQ	241–280 mW/m^2^	[71]
DC-MFC	120	N/A	Uranium-containing wastewater	99.0 U(VI)	269.5 mW/m^2^	[72]
DC-MFC	1000	N/A	Wastewater	92 (Ni); 87 (Cd)	722 mW/m^3^	[73]
DC-MFC	100–200	N/A	Wastewater	90 (COD); 40; 60 (orgN)	1.69 A/m^2^	[74]
DC-MFC	118	30	Sewage sludge	99.08 (P)	~40 mV	[75]
DC-MFC	250	28	Wastewater	95.7; 94.7; 92.37 (COD)	1696.56 mW/m^2^	[76]
DC-MFC	300	30	Wastewater	70–88; 18–44 (COD)	2.2; 44.6; 86.9 mW/m^2^	[77]
DC-MFC	125 (125 cm^3^)	6 (144 h)	Wastewater	99.16 (Cu^+2^)	24.75 mW/m^2^	[78]
TC-MFC	28	~2 (50 h)	Synthetic municipal wastewater	80.0 (Iron); 22.1 (Sulfur)	576.6; 184.8 mW/m^2^	[79]
TC-MFC	28	8	Wastewater	86.2 (Cu^+2^)	420 mW/m^2^	[80]
Stacked MFC	37.5	~9	Barley–shochu waste	36.7 (COD)	15.7 mW/m^2^	[81]
Stacked MFC	28	N/A	Effluent	16.9 (COD)	1023; 1076 mW/m^2^	[82]
Stacked MFC	N/A	N/A	Substrate	N/A	21,111 W/m^3^	[83]
Stacked MFC	N/A	~60	Wastewater	70.0 (Sulfide), 54.6 (COD)	3.29 mA	[84]

SC-MFC, single-chamber MFC; DC-MFC, dual-chamber MFC; TC-MFC, triple-chamber MFC; OrgN, organic nitrogen; COD, chemical oxygen demand; HCQ, hydroxychloroquine.

### 2.2. Electrode Materials

A wide range of materials for electrodes have been used to assess the efficiency of MFCs. Finding efficient materials for the design of MFCs presents great challenges for researchers working in this field. While the operation and implementation of MFC technology have been limited by various factors, the electrodes are the primary aspects of the MFC. Different electrode materials have previously been investigated and tested to enhance MFCs. Fortunately, the test results were encouraging, as the materials showed potential to drive MFC technology forward. In this section, an overview of the different electrode materials that have been used most frequently is provided, as they can be considered to be suitable materials. For an electrode material (anodic or cathodic) to be suitable, it must possess high conductivity, anticorrosive, biocompatible, chemically stable, and high surface area and porosity characteristics, as described in [85]. Using similar electrode materials for the anode and cathode can increase the power density and improve electron transfer by microorganisms.

Table 2 represents the different materials (including advantages and disadvantages) mainly used for the anode electrode. As can be seen from the table, these electrode materials have significant advantages in driving the configuration of MFC technology; despite these advantages, they also face substantial disadvantages. The principal disadvantages of electrode materials are high cost, low surface area, and corrosion issues. Furthermore, most electrode materials (e.g., graphite felt, carbon paper, carbon cloth, and so on) used for construction of the anode electrode can also be used for the cathode electrode. However, the cathode materials should have catalytic properties, high mechanical strength, and high electronic and ionic conductivity, as described in [86]. For example, a Pt-based catalyst is one of the most common cathode materials used in MFC configurations, including oxygen reduction reactions (ORR). This catalyst has significant advantages, such as a low over-potential and large surface area [87], making it a good candidate for process feasibility studies. Nevertheless, the main disadvantages of using a Pt catalyst in the configuration of MFC technology are restricted stability, low availability, and high cost. The high cost of Pt significantly limits the practical applications of MFCs [88], although this electrode material has great potential to improve this technology in terms of performance. More research on Pt-based catalysts is required to reach the goal of reducing cost and moving forward with large-scale applications and commercialization of MFCs.

In addition, an appropriate material allows for the formation of biofilms in the anode region, and the generated bacterial community should remain in contact with the anode surface. However, if the anode material is not suitable (i.e., it is not biocompatible with the growth of the microbes), the electrons may decrease considerably; this would also imply a decrease in bioenergy production, as comprehensively reviewed in [89]. Other crucial factors in electrode fabrication are the anode dimensions, which influence the performance of the MFC. With a large surface, there is a greater chance for microorganisms to grow and respire effectively on the anode surface. This will result in higher electron transfer, increasing the potential of the MFC to generate bioelectricity. Another vital factor to consider is the type of membrane used in an MFC system.

**Table 2:** Advantages and disadvantages of different materials used for the anode electrode.

Type of Materials	Advantage	Disadvantage	Refs.
Carbon paper	High conductivity and stability	High cost and poor environmental friendliness	[90]
Carbon cloth	Great porosity and high conductivity and stability	Small surface area and corrosion issues	[91]
Graphite felt	Great porosity and high conductivity and stability	Low surface area and corrosion issues	[92]
Graphite brush			
Graphite rod	High conductivity and stability, very easy to use, and cheaper	Surface area not easy to increase	[93]
Stainless steel	High conductivity, very easy to use, and cheaper	Easy corrosion, problems with biocompatibility, and very low surface area	[94]
Graphene	High mechanical strength, biocompatibility, and good conductor of electricity	Susceptibility to oxidative environments and expensive and complex to process	[95]
Metal oxide	Low cost, high operating voltage, and ecofriendly	Expensive	[96]
Bamboo charcoal	Low resistance, high mechanical strength, high chemical stability and corrosion resistance, strong biocompatibility, and cheaper	High cost for large-scale implementation	[43]

Yaqoob et al. [97] have concluded that biocompatibility, conductivity, porosity, anode surface area, durability, stability, and low cost of materials are the essential properties that anode electrodes must possess in order to yield good performance. In addition, the authors reported that surface treatment, composites, and coatings are the main aspects of strategies aimed at the modification of anode electrodes.

**Figure 4:**
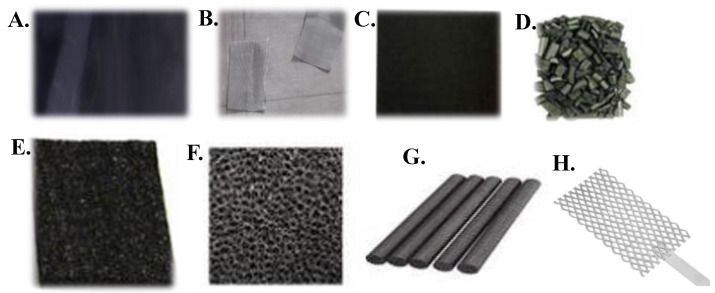
Electrode materials commonly used in bioelectrochemical systems: (**A**) graphite felt; (**B**) stainless steel mesh; (**C**) carbon paper; (**D**) graphite granules; (**E**) carbon felt; (**F**) vitrified carbon cross-linked; (**G**) graphite rod; and (**H**) platinum mesh. Adapted from Yaqoob et al. [97].

Figure 4 shows the most common materials that have been utilized in MFC technology. Notably, each material differs in porosity, biocompatibility and electrochemical stability, surface area, flexibility, and cost. Nonetheless, they all present equally high electrical conductivity. Although these materials have high electrical conductivity, they also present disadvantages such as low resistance (graphite felt), high cost and low surface area (graphite rod and carbon paper), and low bioenergy production (stainless steel mesh). Among the electrode materials, graphite felt can be considered the best material for configuring BESs, as described in [98]. Besides considering the sustainability and environmental impact of BESs, it is also important to consider the practicality of using membranes and electrode reactors when trying to achieve increased economic viability.

### 2.3. Membrane

The membrane is an essential component in the configuration of an MFC. The use of a membrane in an MFC is inevitable, as it prevents the mixture of reactions coming from the cathode and anode compartments; it also prevents the transport of oxygen [99]. In addition, the separator prevents the flow of any unfavorable compound in the reactor, improving the CE (coulombic efficiency) of the MFC [66]. Depending on the variety of membrane, its properties and nature are the determining characteristics that increase or decrease the MFC’s internal load. A high internal load leads to low performance of the MFC and limits its practical real-time application [100]. Despite significant advances in selecting MFC separators in recent years, finding a suitable and low-cost membrane remains a significant challenge for researchers.

**Figure 5:**
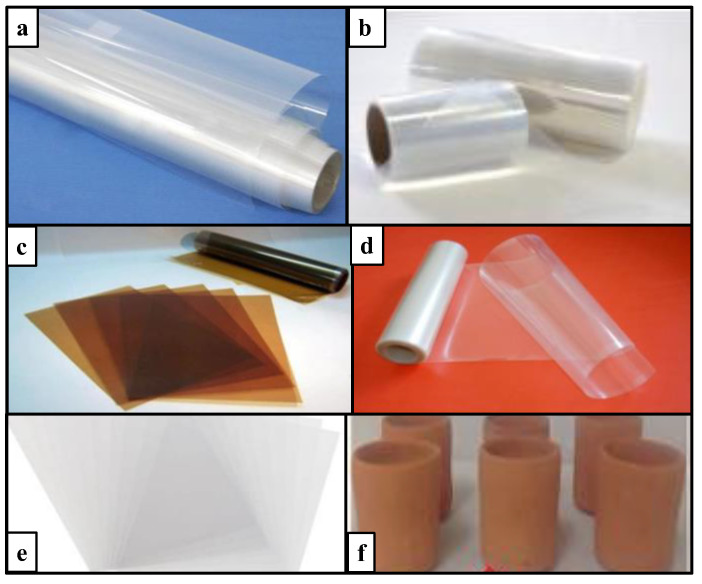
Types of membranes generally used in MFCs: (**a**) Nafion 117 [105]; (**b**) bipolar membrane [102]; (**c**) AEM; (**d**) PEM; (**e**) CEM; and **(f**) clay cup membrane [109].

A variety of separators (membranes) have been used in MFC systems to date (Figure 5). Nafion membranes are commonly used in MFCs, due to their excellent ionic conductivity [101]. Additionally, to replace the Nafion membrane, commercial membranes have been introduced, such as bipolar membranes [102,103], cation and anion exchange membranes (CEMs; AEMs) [104], nanoporous membranes [105], and microfiltration membranes [106]. Several other membranes have been investigated to overcome both economic and environmental challenges [107], such as ceramic membranes [53,108], clay cups [109], and ceramic sticks [10].

### 2.4. Influence of Microorganisms

Microbes are key drivers in the performance of BESs such as MFCs. The microbes are responsible for directly transporting the electrons through oxidation of the substrate. For successful electron transfer to occur, the electroactive microbes must be in contact with the anode surface via the extracellular membranes of the EABs. They (microbes) may also interact with electrodes through nanowires or electron media. In addition, cytochrome proteins from the extracellular membrane are responsible for the electron transfer process [110]. From a predictive point of view, emphasis can be placed on the group of metal-reducing microorganisms, such as *Geobacter* spp. (including *Geobacter sulphurreducens*), which have great potential. Due to their metabolic pathways, *Geobacter sulfurreducens* can produce high current densities in MFCs. Furthermore, *Proteobacteria*, *Pseudomonas*, and *Shewanella* are other groups of microorganisms that have been widely investigated in mediator-less MFC systems [9], as recently discussed in [39]. To understand the electron transfer mechanism on the anode surface of MFCs, it is necessary to understand the model proposed in [111].

Mixed cultures of bacterial communities forming a biofilm (from wastewater) and pure cultures have been widely used in MFCs to increase their working performance regarding power density [112]. However, previous studies have shown that mixed cultures may perform better, as they significantly increase the bioelectricity production in MFCs compared to pure biofilm cultures [113], as has been critically reviewed in [18]. Consequently, continuous bioelectricity production in an MFC using selective or specific microorganisms poses a great challenge. Certain functions may be complex, depending on the habitat of origin of the microorganism. Even so, it is recommended to carry out laboratory Molecular Biology studies, in order to increase the potential of the microorganisms to be used in MFCs. Such studies must be conducted by genetically modifying the genome, which involves the targeted manipulation of bacterial DNA [114]. The genes of importance in the process are the 16S fraction of the ribosomal RNA and the DNA of the plasmids. As a result, a strain with more significant bioelectricity generation potential may be obtained. Another alternative would be to use extremophile organisms that adapt to any operating condition without being affected by external and internal factors during the investigation. One of the advantages of using these organisms in MFCs is that they can produce bioenergy from any biodegradable substrate.

## 3. Bioenergy Production from MFCs

### 3.1. Bioelectricity Production

**Figure 6:**
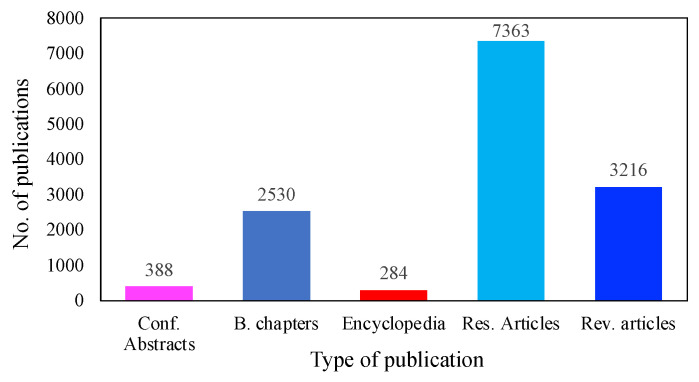
Publications on MFCs during the last decade (source: ScienceDirect database; 4 June 2023).

MFC technologies have received significant attention, due to their extraordinary capacity to produce bioenergy without harming the environment. Figure 6 illustrates the advances that have been made to bring MFC technology into the realm of practical application. To date, more than 7363 investigations have been conducted on the use of MFCs for low-cost electricity generation. In addition, over 3216 review papers have been published on different aspects of MFCs, followed by 2530 book chapters, 388 conference abstracts, and 284 encyclopedias. It is worth mentioning that these data were obtained only from the ScienceDirect database, as there were more publications on energy recovery using MFC systems than on other platforms.

Great strides have been made in improving bioelectricity production by MFCs. Many recent studies have reported high power generation efficiency using MFCs inoculated with different types of waste (e.g., wastewater, sludge, and human urine). Table 3 details the progress and advances of MFC technologies as of 2022. For this table, some of the most promising studies on the configuration of MFCs and the critical parameters in the energy production process were selected.

The highest power density of 2203 mW/m^2^ was obtained using an SC-MFC inoculated with anaerobic sludge as an outstanding inoculum [115]. The SC-MFC was constructed with a graphite brush as an anode, while modified materials—that is, graphite-based nanomaterials and platinum/carbon (Pt/C)—were utilized for the cathode. The performance achieved in the study was referred to as the cathode modification and the operating conditions of the MFC (i.e., 30 days of operation and 27 cycles). In contrast, Subran et al. [116] obtained a highest power density of 590 mW/m^2^ (with 78% COD removal efficiency) in an SC-MFC using carbon cloth as an anode and cathode, separated by a Nafion 117 membrane. This value was almost four times lower than that reported with Pt/C [115]. In the study by Sathish et al., rGOHI-AcOH (acetic acid) and rGO/Ni (reduced graphite oxide nickel nanoparticle composite)—two reduced graphene oxide hydrogen iodides—were used with Pt/C as catalysts [115]. The results were interesting, as they indicated that the rGOHI-AcOH-based catalysts, as mentioned earlier, could feasibly drive the MFC system without interruption (in terms of power density). The power output found in [116] was 76.6% higher than the maximum power output (138 mW/m^2^) reported in a tubular MFC (T-MFC) [117]. The T-MFC was fabricated using a graphite rod as an anode electrode, carbon cloth-coated Pt (200 cm^2^) as a cathode electrode, and nanocomposite as a proton exchange membrane. The findings indicated that the best option for improving the performance of an MFC is modifying its electrode (i.e., synthesized and characterized nanocomposite membranes) and using suitable configuration materials. In the previous study by Bensaida et al. [118], the highest power density of 833.33 mW/m^2^ was obtained in a prototype MFC inoculated with Mg(OH)_2_-coated iron nanoparticles. This value was 29.19 and 83.34% superior to that in agro-waste and synthetic wastewater, respectively; however, it was approximately three times inferior to that reported in anaerobic sludge elsewhere [115].

Anaerobic sludge appears to be the most suitable inoculum for use in MFCs to improve the power density efficiency, compared to others. This kind of inoculum (sludge) has tremendous potential to increase the performance of MFC systems [115]. This performance enhancement was due to the proper characteristics related to the sludge used, including operating conditions and modification of the reactor. It is crucial to note that the efficiency of an MFC also depends on factors such as the type of electron acceptors and catalysts used. Both electroactive biofilms and extracellular electron transport in MFCs are highly dependent on the physicochemical properties of the operational anode surface; therefore, surface modification of conventional anode materials is critical for improving the performance of MFCs. However, more studies on the use of substrate and electrode modifications are required in order to reliably increase energy production in MFC technologies.

On the other hand, in their quest to increase the amount of OM and keep bacterial populations active on the anode surface, researchers have investigated other types of substrates with great potential, such as acetate, butyrate, glucose, cellulose, and sucrose. These substrates have all been used in MFCs in an attempt to improve their output power, as described in [2]. The main idea of using such substrates in MFCs is to improve the transport of protons from one compartment to another, in addition to forming good biofilms on the electrode and keeping the bacterial community active. The highest power densities in the range of 305 and 506 mW/m^2^ have been recorded in an SC-MFC inoculated with butyrate (1000 mg/L) and acetate (800 mg/L), respectively [119]. It was noted that the SC-MFC using acetate presented better performance, in comparison to that inoculated with butyrate. The difference between the reactors regarding the power density arose from the concentrations of the substrates. Later, in an SC-MFC inoculated with glucose, the highest power density of 52 mW/m^2^ was reported, approximately 9 times and 5 times less than those derived for butyrate and acetate, respectively [120]. Hashmi et al. [121] have recently reported the highest power density of 71.12 mW/m^2^ in a DC-MFC when removing hazardous materials from wastewater. This yield in MFC was due to the addition of 350 μmol/L of C_6_N_6_FeK_3_ (potassium ferricyanide) in the cathode compartment. Concurrently, 180 μmol/L of CH_2_ (methylene) was applied to the anode compartment. As mentioned above, a high concentration of the chemical compounds is an excellent option to increase the performance of MFCs while treating wastewater.

According to the above studies, modifying the electrode material is the better or cheaper option to improve MFC performance, in terms of power density, compared to conventional electrodes. The configuration of an MFC using a conventional electrode is expensive, making its large-scale application much more costly. Furthermore, another alternative to improve the performance of MFCs is the use of organic substrates, which are excellent electron donors. Finally, anaerobic sludge has been reported to be one of the best inoculums (due to characteristics such as bacterial diversity, as described earlier) for improving power generation in MFCs, followed by Mg(OH)_2_-coated iron nanoparticles, rGOHI-AcOH, and acetate as excellent substrates (with excellent carbon sources to improve or stimulate EAB) for MFC technologies [115,119]. Furthermore, algae have been reported to be a good substrate for enhancing the performance of MFCs for the recovery of value-added products from wastewater [122,123], including biofuels [124], bioremediation, and bioelectricity generation [125,126]. According to the literature, MFC-based algae have great potential for improving power efficiency, nutrient removal, heavy metal recovery, and bioremediation of contaminants.

Yaqoob et al. [127] recorded the highest current density of 36.84 mA/m^2^ (within 20 days of operation) using potato wastewater as an electron donor for the biodegradation of pollutants in a benthic microbial fuel cell (BMFC). This MFC performance was 82.28% less than that previously reported in a DC-MFC operated with potato waste as the substrate [128]. The study by Yaqoob et al. [127] demonstrated the simultaneous effective removal of OM and COD (up to 84%) and bioremediation of pollutants (up to 90%). The electricity generation behavior observed in both studies was due to the configuration materials used to assemble the electrodes. Furthermore, the characteristics of the substrates, such as chemical composition (e.g., pH; electrical conductivity, EC; and so on), as well as the operating conditions, were factors that influenced the performance of the MFCs in both studies. For example, in the study by Yaqoob et al. [129], the pH and EC of the substrate before application were 7.04 (neutral) and 59 μS/cm (increased by 26% after treatment), respectively, which improved the production of power in the BMFC. The neutral pH of a substrate (or environment) facilitates the development or growth of microorganisms, promoting optimal activity levels on the anode surface [130]. In addition, OM concentrations and the bacterial community play critical roles in improving the performance of MFCs.

Furthermore, in another investigation, an SC-MFC was configured using bamboo charcoal (BC) for the anode electrode and a Pt-coated carbon cloth for the cathode [43]. The working volume of the MFCs was 530, 530, and 500 mL, and they were inoculated with potato-processing wastewater. In one MFC, a maximum current density of 1140 mA/m^2^ was observed. The current density achieved by Sato et al. [43] in potato waste-fed SC-MFC was higher than that in the above studies. This performance could be due to the physicochemical parameters of the substrate (potato waste) and the high EC and low resistance when BC is used as an anode electrode. Other key characteristics of BC include its biocompatibility, high chemical stability, and mechanical strength [43].

In summary, the above findings indicated that potato waste—like other organic substrates—is potentially an excellent alternative, allowing for enhanced power generation and wastewater bioremediation performance when using MFCs. Furthermore, the electrode materials are other aspects to be considered when implementing MFC technology. However, more investigations are required to evaluate the influence of potato wastewater in MFC technological development.

**Table 3:** Summary of production of bioelectricity using different MFC configurations.

Configuration Type	Electrode Materials		Membrane Type	Substrate	Working Volume (mL)	Operation (Days)	Max. Power Generation	Refs.
	Anode	Cathode						
SC-MFC	Carbon felt (16 cm^2^)	Carbon felt (31 cm^2^)	Clayware	Synthetic wastewater	150	30	995.73 mW/m^3^	[131]
DC-MFC	Carbon fiber	Carbon fiber	SPEEK-goethite	N/A	N/A	N/A	73.7 mW/m^2^	[132]
T-MFC	Graphite rod	Carbon cloth coated Pt (200 cm^2^)	Nanocomposite	Sewage wastewater	300	3 weeks	138 mW/m^2^	[117]
DC-MFC	Graphite	Graphite	Nation 117	Activated strains	500	N/A	12.82 mW/m^2^	[133]
SMFC	Carbon-polymer composite	Carbon cloth (3 × 3 cm^2^)	N/A	Sediment from wastewater	100	30	1056.6 W/m^3^	[134]
MFC	Carbon fiber brushes	Carbon fiber brushes	Nafion 117	Glucose, yeast, and MB	800	N/A	5.55 W/m^3^	[135]
SC-MFC	Carbon brush	Lignin-derived activated carbon	N/A	Sludge	125	N/A	6.7–6.5 mW	[136]
C-MFC	Activated carbon coated carbon veil (30 mg/m^2^)/pressed over stainless steel mesh	Activated carbon coated carbon veil (30 mg/m^2^)/pressed over stainless steel mesh	Flat terracotta membrane (12.25 cm^2^)	Human urine and sludge	12.5	N/A	492.85 μW	[32]
S-MFC	Graphite felt (7 × 7 × 0.4 cm)	Carbon cloth coated-Pt, plain carbon cloth, and graphite felt	N/A	Soil	N/A	~50	87.3 mW/m^2^	[137]
DC-MFC	Graphite filter	Stainless steel mesh	Carbon-ceramic composite	Wastewater	5.3 (cm^3^)	N/A	0.699 W/m^3^	[138]
SC-MFC	Wired stainless steel 60 mesh	Wired stainless steel 60 mesh	Cylindrical terra- cotta pots	Textile effluent	N/A	N/A	21–42 mW/m^2^	[139]
SC-MFC	Graphite brush	graphite-based nanomaterials	N/A	Anaerobic mud	N/A	30	2203 mW/m^2^	[115]
SC-SMFC	Unidirectional Carbon Fiber (total area 81 cm^2^)	Unidirectional Carbon Fiber (total area 40.5 cm^2^)	N/A	Marine and fluvial sediments	2000	30	70 mW/cm^2^	[140]
SC-MFC	Graphite felt (thickness 10 mm, diameter 80 mm)	Graphite felt (thickness 10 mm, diameter 80 mm)	N/A	Oily sludge	2000	~31	1277.90 mW/m^3^	[141]
SC-MFC	Carbon cloth	Carbon cloth	Nafion 117	Agro-waste	200	N/A	590 mW/m^2^	[116]

T-MFC, tubular MFC; MB, methylene blue; C-MFC, ceramic-based MFC; S-MFC, soil MFC; SC-SMFC, single-chamber sediment MFC.

### 3.2. Other Types of Energy Production

**Figure 7:**
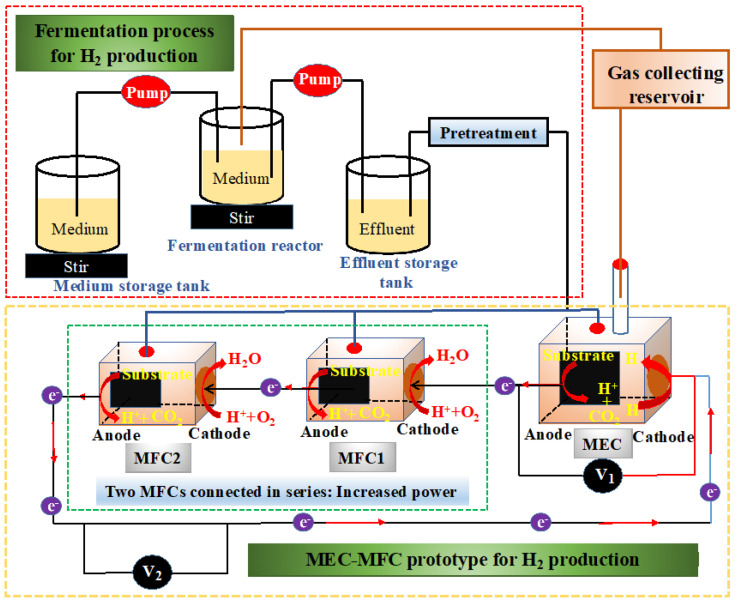
Diagram of H_2_ production through a dark fermentation process. As can be seen, a prototype MEC-MFC is coupled to improve the power generation during the process. Adapted from the combination of references [29,142].

In recent years, great strides have been made regarding the use of BESs, such as MFCs, for the simultaneous treatment of wastewater and production of fuels. This progress has been achieved by combining BES prototypes to improve their process performance. In this line, the use of MFCs for the production of both CH_4_ and H_2_ has captured the attention of researchers worldwide. Apart from the fact that this technology is environmentally friendly, it is also an excellent option for producing fuels while eliminating pollutants in wastewater. Furthermore, MFC technologies are less expensive, when compared to conventional methods. Figure 7 depicts the dark fermentation process for production of fuels such as H_2_.

Hao et al. [143] have recently constructed an SC-MFC (working volume of 2000 mL) coupled with an anaerobic membrane bioreactor (AnMBR) to enable enhanced CH_4_ production. While 40 cm^2^ of carbon felt was utilized as an anode, the cathode was constructed from various materials (e.g., non-woven carbon cloth, platinum carbon, and carbon black). Before assembly of the SCMFC-AnMBR, the carbon felt was sterilized using 1 mol/L HCl and NaOH. The SCMFC-AnMBR was inoculated with synthetic wastewater (SWW) and compared to the conventional one (C-AnMBR). The SWW was added to SCMFC-AnMBR with a concentration ratio of 205:5:1 (C:N:P), and the main components of SWW were: (1) 3000 mg/L of glucose (C_6_H_12_O_6_), (2) 120 mg/L of monopotassium phosphate (KH_2_PO_4_), (3) 120 mg/L of ammonium chloride (NH_4_Cl), (4) 112 mg/L of iron chloride (FeCl_2_), and (5) 30 mg/L of magnesium sulfate (MgSO_4_). The SCMFC-AnMBR indicated high recovery efficiencies of 38.89 mg/L (COD) and 67.84 mg/L (CH_4_) and presented a maximum voltage of 107 ± 14 mV. This study demonstrated that the SCMFC-AnMBR is better than the conventional system, as it was stable in terms of the micro-bioelectric field environment [143]. Meanwhile, in a previous study [144], a submersible MFC (SMFC) coupled with anaerobic digestion (AD) was configured for enhanced CH_4_ production. The prototype SMFC-AD was inoculated with glucose at different concentrations (2, 4, and 10 g/L). The authors compared the performance of the combined system and the single one (AD). The results indicated that, at a concentration of 2 g/L of glucose, the SMFC-AD reached a higher CH_4_ production of 320 mg/L (COD; 0.32 g/L COD), when compared to the other concentrations used in this study. The CH_4_ production achieved [144] was 8 and 5 times higher than that reported when using the SCMFC-AnMBR. According to this study’s findings, it can be argued that the best glucose concentration to enhance CH_4_ production is 2 g/L. Furthermore, high glucose concentrations negatively affected the reactor’s performance, in terms of fuel production.

Additionally, in a previous study by Nguyen et al. [145], single-chamber microbial electrolysis cells (MECs) using a simultaneous dark fermentation (DF) process (Figure 7) were constructed. The DF was combined with MECs to form the so-called DF-MEC. Then, the DF-MEC was inoculated with a species of algae called *Saccharina japonica* (sDFMEC), in order to improve fuel production and accelerate the process. The sDFMEC recorded a maximum production of H_2_ of 438.7 ± 13.3 mL/g-TS, outperforming the control (i.e., DF-MEC). At the same time, the sDFMEC system reached maximum CH_4_ production and COD removal of 63.1 ± 3.4 mL/g-TS and 75.6 ± 1.4%, respectively. Nonetheless, DF-MEC showed an efficient recovery of 32.2%, achieving a maximum H_2_ production of 403.4 mL/g-TS [145]. In contrast, in the study by Gebreslassie et al. [146] a maximum H_2_ production of 110 mL/g-VS was reported in a similar sDFMEC. This yield was 3.66 times lower than that reported by Nguyen et al. Later, in an sDFMFC constructed using a surface-modified stainless steel mesh cathode, a higher H_2_ production of 408 mL/g-TS was recorded [147]. The authors modified this system to optimize the electrode performance during the DF process. They revealed that anodization (i.e., coating an oxide layer over a metal to make it corrosion-resistant) is the best alternative for improving fuel production using bioelectrochemical systems. These findings indicate that sDFMEC is more effective than the combination of SCMFC-AnMBR for fuel production, due to the significant effect of adding *S. japonica* into the sDFMEC reactor. However, all of these methods can feasibly improve CH_4_ and H_2_ production while conducting bioremediation of contaminants from wastewater.

Other strategies used to improve fuel production (i.e., H_2_ and CH_4_) include the use of other types of substrates, such as biochar, which led to increases in H_2_ production of up to 118.5% and in CH_4_ of up to 14% [148]. Calcium lignosulfonate-based biochar led to a 50% increase in H_2_ [149], a biochar catalyst increased H_2_ by 52% [150], and electroactive cultures (EACs) have also been tested. More recently, a DC-MFC was constructed to evaluate the effect of CH_4_ injection on anaerobic oxidation (AO)-coupled MFCs (AO-MFCs), in terms of power generation [151]. Two EACs (acetate and formate) were used in the AO-MFCs, in order to improve the performance of the fabricated prototype. For start-up of the system, 84.5 cm^2^ (13 × 6.5 cm) of breathable cloth was used as an anode, while the cathode was made of 81 cm^2^ (9 × 9 cm) of carbon felt, which were separated by an ultrafiltration membrane. The anode chambers were inoculated with 40 mL (0.04 L) of anaerobic sludge. The obtained results indicated that the specific EACs were responsible for converting CH_4_ to CO_2_ while producing bioelectricity.

Liu et al. [152] have constructed a novel prototype MEC coupled with AD (MEC-AD; working volume 400 mL) operated with few-layer Ti_3_C_2_TX MXene (FL-MXene), multi-layer Ti_3_C_2_TX MXene, and MAX phase titanium aluminum carbide (MAX). Subsequently, the reactor was inoculated with cattle manure (CM) and sewage sludge (SS). Furthermore, graphite rods were used for the anode and cathode materials. The MEC-AD operated with a 0.035 wt% of ML-MXene showed a maximum CH_4_ production of 358.7 mL/g VS. This result was superior to that reported in MECs (38.4 ± 1.7 mL/g TS) elsewhere [145]. The recorded difference was due to the systems having been configured differently and operated under different environmental conditions. Table 4 summaries the performance obtained in various BES studies, in terms of H_2_ and CH_4_ production.

## 4. Applications and Challenges of MFCs

MFCs have been explored for their real-time application, similar to other bioelectrochemical systems. The main idea of research in this field is the scaling up of MFCs toward their commercialization. MFCs play a fundamental role in the bioremediation of contaminants, such as azo dyes, heavy metals (e.g., Pb, Cr, Hg, and Cd), sulfides and sulfates, nutrients (nitrates and phosphates), antibiotics, and petroleum, from wastewater and soil [158,159,160,161]. In this context, significant progress has been made with respect to MFC applications. Typically, MFCs were designed at a laboratory scale to produce bioelectricity using microbes in a bioreactor [13]. As the results were encouraging, researchers began to further investigate biological systems such as MFCs. However, shifting MFCs from laboratory scale to practical applications faces significant challenges worldwide [162].

**Figure 8:**
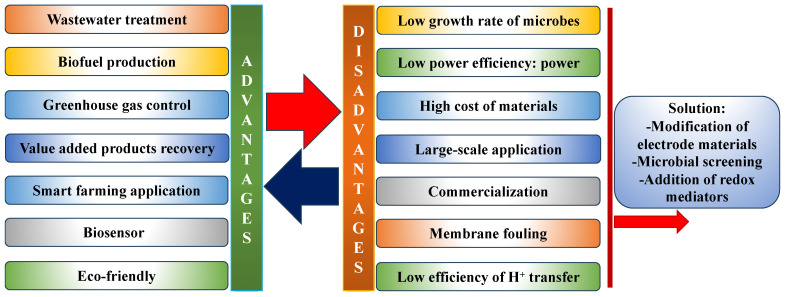
Pros and cons of MFC technologies.

Furthermore, the challenges faced by MFC technologies include low power density, the high cost of electrode materials, continuous electricity generation, low conductivity of electrode materials, and membrane fouling, as described in [29]. In contrast, MFC is a biotechnology with significant advantages (Figure 8). Studies have reported that the low efficiency of MFCs may be due to their over-potential (internal resistance), which depends on the system configuration [163,164]; in addition, this could limit the power generation in terms of power density [165]. Other significant challenges are electrode contamination and aging over time. It has been previously suggested that modification of the electrode materials and reactor configurations, as well as the use of suitable substrates, in order to optimize the performance of MFCs may reduce their internal resistance [166]. In addition, the choice of microorganisms with great OM oxidation potential for electron transfer is another aspect that should be taken into consideration during the configuration of MFCs [164]. Furthermore, using a catholyte (e.g., potassium dichromate, K_2_Cr_2_O_7_ or potassium permanganate, KMnO_4_) or anyolite (e.g., bacterial culture or growth media) in an MFC could be an excellent alternative to improve its power density, as described in [167]. Human urine [168,169,170], livestock urine (from sheep, goats, and cows) [16], and potato waste [171] have recently been used in MFCs and have been confirmed as excellent feedstocks. The power densities were significantly increased after adding the above substrates to the MFCs. These feedstocks are suitable to enhance the problem of low power density in MFC technology, potentially promoting its real-time application. Recent studies have demonstrated the feasible real-time application of MFCs to power devices such as BOD biosensors [172], the Internet of Things (IoT) [173], and digital clocks and LEDs [10], as well as to charge cell phones [174]. Nevertheless, more studies are needed to carry MFCs toward the realization of real-time application and commercialization.

In summary, the application of MFCs in different fields, including wastewater treatment, bioremediation of pollutants, and nutrient recovery while producing bioelectricity, is still in its beginning stage. Thus, there is a need for more research on the scaling of MFCs from laboratory to practical application, despite their limitations in terms of scaling up due to membrane fouling, voltage losses, and low power output. In addition, the knowledge gaps in terms of the designing, configuring, and operating conditions of MFC technologies should be addressed as soon as possible. It is worth mentioning that more studies on different designs of prototype MFCs are required, in order to carry out the design of such systems for large-scale and commercial applications. In previous critical reviews [93,175], several potential solutions have been suggested to overcome the limitations of MFC technology. Furthermore, there is a need to determine where MFC systems could be used as a stand-alone technology or as part of a combined system. Further work is required to understand appropriate models for the implementation of MFCs as part of a broader sustainable electricity production strategy, which should be based on novel designs and prototypes to convert biowastes into bioelectricity. Finally, based on the above, further work can be suggested with respect to evaluating the capacity and feasibility of MFC technologies.

## 5. LCA of MFCs

BESs such as MFCs have been a key area of research, regarding their commercialization worldwide, due to the high global energy demand and accelerated population growth and industrialization. The literature has reported that 80% of the world’s energy generation depends mainly on the exploitation of fossil fuels [176]. However, these energy sources produce high greenhouse gas (GHG) emissions [177], negatively affecting the environment. As such, the use of MFCs to produce electricity is safer than conventional technology. Nevertheless, these systems require an in-depth evaluation of their environmental impact and techno-economic feasibility before implementation. More information on this line is available in [178].

As MFCs are devices that recycle waste materials for energy generation (e.g., electricity and hydrogen) and improve water quality, they have great promise to become feasible bio-economic systems; however, wastewater substrates containing buffer salts (e.g., phosphates and carbonates), either in the anode or cathode of the system, are not economically or environmentally practicable [179]. Additionally, besides considering the sustainability and environmental impact of BESs, it is also important to consider the practicality of using membranes and electrode reactors when trying to achieve increased economic viability.

LCAs are an emerging approach used to assess both bioenergy production and carbon sequestration through all the stages of a product’s life cycle. Additionally, an LCA is a well-known standardized technique that systematically and quantitatively evaluates the potential risks and impacts of a product or system’s environment throughout its life cycle. According to the International Standards Organization (ISO 14040: 2006), an LCA includes the following stages: (i) goal and scope definition, (ii) life-cycle inventory analysis (LCI), (iii) life-cycle impact assessment (LCIA), and (v) interpretation of results [180]. The four stages mentioned above should be involved in studies on the MFC LCA, considering the type of electrode materials used and the energy consumption involved. In terms of material assessment, an effective LCA process must cover all energy and emissions impact stages. In the case of MFC configuration, it must be understood that the materials used should be entirely recyclable or biodegradable and, thus, environmentally friendly. The first stage of the MFC LCA (i.e., goal and scope definition) defines the goals, purposes of the assessment, and the decisions made at the establishment of the experiment, including the type of configuration (e.g., SC-MFC or DC-MFC), the choice of material of fabrication (e.g., PEM, activated carbon or stainless steel), operating time or operational stages, and the functional unit (FU; i.e., the type of substrate/wastewater treatment). Subsequently, in the second phase of the LCA method (LCI), the use of materials and chemicals, effluent emissions, energy generation, and consumption (profitability) should be evaluated before construction and implementation of the MFC. In a previous LCI [181], which reported on the configuration of an SC-MFC (1 L of wastewater treated) consisting of an air-cathode with carbon cloth (7.9 × 10^−4^/FU) and carbon paper (5.89 × 10^−4^ g/FU) as an anode electrode [66], the energy consumption was 2.4 × 10^−3^ kWh/FU (profitability of 1.68 × 10^−5^ kWh/FU) [66,181]. In contrast, the energy consumption for the configuration of a DC-MFC was 3.34 × 10^−3^ kWh/FU when using graphite felt as an anode and carbon brush as a cathode material [181,182]. In turn, concerning the release rate of environmental contaminants from effluent in the form of COD, the DC-MFC proved to be the best option, yielding 43.5 mg/L/COD [66], compared to the SC-MFC with the air-cathode, which presented 112.5 mg/L/COD [182]. According to the above, it can be argued that the LCI is responsible for determining the potential environmental impacts during the LCA process, as has been described elsewhere [178].

Furthermore, the third stage of the LCA (LCIA) involves evaluating the environmental impacts of MFC technology during its operational stage. This could include simultaneous wastewater treatment and electricity production. The LCIA comprises two steps: (1) midpoint impact categories, such as the source of emissions and resources [181]; and (2) endpoint indicators, which focus on the damage categories, as reviewed in [180]. For example, during such an assessment, the following parameters can be considered: global warming potential, human health, ecosystems, air, and water qualities [178], as well as the impact of the technology on soil quality, which could be beneficial for sustainable agriculture [183]. Finally, the interpretation of the results is the final stage of the LCA, which involves the operation of the MFC system, ensuring that both the LCA method used is suitable and corresponds to the first stage, and that the overall environmental loads of MFCs are significantly low when compared to conventional systems. The interpretation of the results during the LCA is focused on the reactors/MFCs that present the highest environmental loads. For example, a high environmental load on an MFC may occur when it has low treatment capacity due to its high hydraulic retention time (HRT). However, HRT is crucial in improving the efficiency of MFCs with respect to outpower [184]. In this context, LCIA data indicated that the energy consumption to treat 1 L of wastewater using an SC-MFC and a DC-MFC was 3.32 × 10^−9^ and 4.62 × 10^−9^ kWh/FU, respectively. These values represent the impact category during the LCIA study (e.g., reflecting global warming or human health) [181].

Zhang et al. [185] have developed LCA models to investigate the ecological impact of three different BESs: MEC, MFC, and MDC systems. The MEC was found to have the best performance and lowest negative environmental impact, compared to the MFC and MDC systems. This is largely a consequence of the production of H_2_O_2_ (hydrogen peroxide) in the MEC. Nevertheless, the low power generation of 10 W m^−3^ (volumetric power density) recorded in both the MFC and MDC systems was correlated with the external operating conditions, as described in [186]. Low levels of substrate remediation led to the accidental leakage of high COD water, membrane fouling, and the release of unidentified microbes. Another study has also reported a proportional relationship between the change in environmental impacts and the power density generated by BESs [185]. This research suggests that, in order to achieve positive environmental impacts in MFCs and MDCs, higher power density production is required.

In another LCA study, Zhang et al. [187] assessed the environmental impacts of an osmotic microbial fuel cell (OsMFC), which used a forward osmosis (FO) membrane integrated into a conventional MFC to improve the recovery of high-quality water and power generation. The findings revealed that the OsMFC prototype had higher global warming potential than other MFCs, due to the use of materials in its configuration such as stainless steel in the cathode electrode and a polymethylmethacrylate plastic sheeting. The above studies demonstrated how the appropriate coupling of hybrid BESs could make them feasible (i.e., economically viable) while also reducing their environmental impact. On the other hand, an appropriate system configuration coupled with suitable, low-cost materials can profoundly affect the environmental impact of a BES. Corbella et al. [188] have demonstrated that a constructed wetland MFC (CW-MFC) coupled with a graphite-based anode reduced the environmental impact of these technologies by 20%. Such a reduction indicated the most essential environmentally friendly alternative to replace conventional CW systems. However, this system was more expensive (by 1.5 times) than the traditional CW system, which researchers must re-evaluate before practical use. These findings indicate that choosing suitable anode materials in the configuration of the MFC system is advantageous, due to their economic feasibility and environmental footprint.

On the other hand, an auto-circulating bioelectrochemical reactor (AutoCirBER) was designed at the laboratory scale to treat 8.4 L of DWW continuously [189]. An experimental LCA was performed on the AutoCirBER system, demonstrating its environmental practicability in the long term (10 years); moreover, this system presented more than 90.4% COD removal and 59.55 W.h net annual energy recovery. For real-world applications, it is important to conduct further studies that include cost–benefit analyses, fluid dynamic analyses, and so on [189]. According to Pant et al. [190], for the implementation of BESs (e.g., MFCs) at a commercial scale, it is essential to conduct a complete LCA including (i) the choice of the functional unit, (ii) an appropriate system boundary, and (iii) co-products [190]. Finally, an LCA can provide significant insights to renewable energy policymakers and WWT stakeholders regarding the environmental impacts of various MFC systems [181].

The environmental impact assessment of MFCs is essential, which has received much research attention compared to other aspects, such as the design, configuration, operation, and performance evaluations [190]. Previous studies have focused on evaluating the negative impacts of these systems on the environment and human health; however, more work in this context still needs to be completed. The lack of LCA studies is even more evident in the case of MFCs, as a sustainable approach to replace the accelerated exploitations of fossil fuels. However, a few exceptions can be noted. For instance, one study by Shemfe et al. [191] has compared the environmental impacts of conventional systems and MFC technology, concerning their potential to treat wastewater while simultaneously producing bioelectricity and recovering value-added products. In addition, it was demonstrated that the LCA of BESs such as MFCs allows researchers to understand the performance of such systems in terms of wastewater treatment (WWT). Furthermore, in one study, an LCA provided the main advantage of avoiding fossil-based formic acid (HCOOH) production (up to 93%), while power generation for WWT (12%) for the system outweighed the corresponding costs of the BES (−5%), giving a net energy savings equivalent to 61 MJ kg^−1^ HCOOH [191].

According to the discussion above, both techno-economic assessments and LCAs may be conducted to determine the economic viability and environmental impact of emerging technologies such as MFCs during their implementation. Before its use, every emerging technology must be evaluated in terms of GHG emissions (e.g., CO_2_ and CH_4_). Thus, using an LCA allows researchers to assess the environmental impact of MFCs and propose possible solutions to mitigate the potential risks associated with implementing the technology [192].

In summary, the LCA of MFCs is a good tool for evaluating the environmental impacts and sustainability of different materials, such as electrodes and membranes. It is necessary to choose electrode materials with lower environmental impact when developing MFC systems. The electricity produced during the operation of an MFC is tiny, compared to the energy consumption, which could reduce the ecological benefit of the MFC. Nonetheless, further large-scale investigations using large-volume MFCs are required to assess the benefits that may be brought with respect to electricity production during the implementation of MFCs. The scaling-up of MFCs could be an excellent alternative to improve their environmental performance.

## 6. Conclusions and Future Research Directions

MFCs have become promising self-sustainable and clean energy sources utilizing many different substrates (organic sources), which have mainly been used for wastewater treatment. MFCs have great potential to produce bioelectricity while treating wastewater through activated sludge, as well as removing pollutants. Nevertheless, these systems have faced significant limitations and challenges since their implementation. The major limitations of MFC technologies are high internal resistance, unstable current, biofouling, low power output, and mass transport loss, and the primary challenges faced by MFC researchers include expensive electrode materials, scaling up to large volumes, and energy losses due to the resistance of the conducting materials when compared to conventional systems. It will be necessary to address these challenges in order to overcome the limited real-time application and commercialization of MFCs. Despite these limitations, MFCs present various advantages, such as the ability to power BOD biosensors, digital clocks and LEDs, and sensors integrated into the Internet of Things (IoT).

Furthermore, MFCs have a significant advantage in terms of reducing important greenhouse gas emissions. Hence, it is necessary to perform an LCA of MFCs in order to mitigate their environmental impacts. On the other hand, idealized design and operating conditions, such as the smaller electrodes, reactor volumes, and static conditions commonly used in laboratory-scale setups, often result in better performance than in real-time large-scale application systems. Further research is required to address the limitations and challenges of MFCs, including scaling up using modified electrode materials, which may make this technology practicable and commercial.

## Figures and Tables

**Table 4:** Summary of H_2_ and CH_4_ production using different BESs.

Type of Reactor	Applied Energy	Production Rate		Refs.
		H_2_	CH_4_	
MEC-MFC (units in series)	7.473 × 10^−4^ A	7.32 mL/d	N/A	[153]
MEC-MFC (units in series)	6.631 × 10^−4^ A	6.50 mL/d	-	[153]
MEC-MFC (units in parallel)	4.062 × 10^−4^ A	3.98 mL/d	-	[153]
MEC-MFC (units in parallel)	3.849 × 10^−4^ A	3.77 mL/d	N/A	[153]
sDF-MFC	N/A	0.21 L/L/d	N/A	[146]
sDF-MFC	-	0.34 L/L/d	-	[146]
sDF-MEC	0.8 V	438.7 mL/g-TS	N/A	[145]
DF-MEC	0.8 V	403.5 mL/g-TS	-	[145]
sDFMEC	0.8 V	492.3 mL/g-TS	N/A	[154]
BES/MEC	N/A	N/A	77.13 L/kg	[155]
CW-MFC	0.27 W m^3^	N/A	9.5 mg/m^2^/h	[156]
MFC-MEC	0.7 V	N/A	0.354 mL/h/L	[157]
MEC	0.8 V	1.22 L/L/d	-	[147]
sDF-MEC	0.8 V	0.45 L/L/d	-	[147]

MEC-MFC, microbial electrolysis cell coupled with a microbial fuel cell; MEC, microbial electrolysis cell; sDF-MEC, simultaneous dark fermentation microbial electrolysis; DF-MEC, dark fermentation microbial electrolysis; sDF-MFC, simultaneous dark fermentation microbial fuel cell; CW-MFC, constructed wetland microbial fuel cell.

## Data Availability

Not applicable.
